# Supplementary material for: Maternal inheritance of functional centrioles in two parthenogenetic nematodes
Source: Nat Commun. 2024 Jul 18;15:6042. doi: 10.1038/s41467-024-50427-5 (PMC11258339; doi:10.1038/s41467-024-50427-5)
Supplement: Supplementary file 1 — Supplementary Information [file 41467_2024_50427_MOESM1_ESM.pdf]

Supplementary Fig. 1

a

	RSPKR3021.PRJEB1297.WBPS18	Our assembly
Number of contigs	471	5
Largest contig	7,271,431	11,164,410
Total length	47,265,539	44,838,991
N50	537,195	9,292,632
L50	22	3
GC (%)	32.04	32.18
Merqury Quality Value	-	44.7485
HapPY haploidy score	-	0.98

b

	Ortholog set	Scores in BUSCO format
RSPKR3021.PRJEB1297.WBPS18	Metazoa	C:68.6%,F:3.8%,M:27.6%
Our assembly	Metazoa	C:68.5%,F:3.7%,M:27.8%
RSPKR3021.PRJEB1297.WBPS18	Nematoda	C:64.8%,F:2.3%,M:32.9%
Our assembly	Nematoda	C:65.0%,F:2.3%,M:32.7%

c

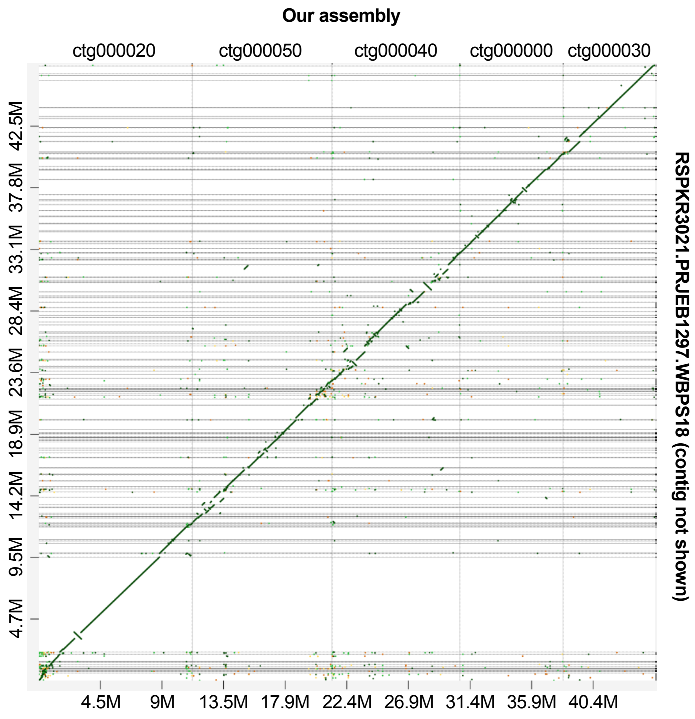

d

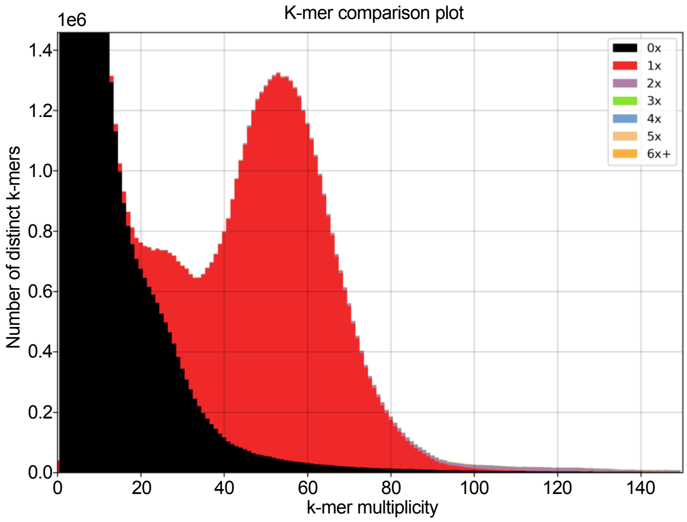

e

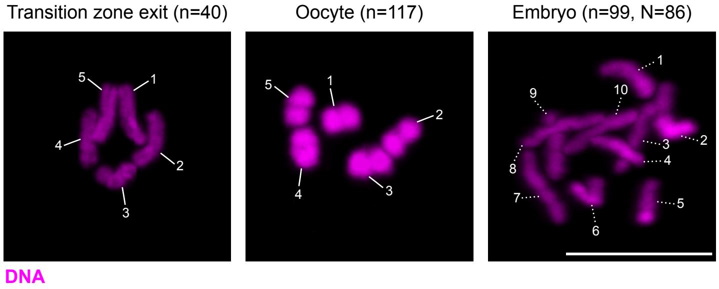

Supplementary Figure 1: Chromosome-scale genome assembly and transcriptome of *R. diutinus*. Genome assembly comparison between the previously published *R. diutinus* genome[18] and our chromosome-scale assembly. **(a)** Main characteristics of the two genome assemblies. **(b)** Summary of BUSCO genomic data quality assessment results. **(c)** D-Genies dot plot comparing the two *R. diutinus* genome assemblies. **(d)** KAT 27-mer comparison between the PacBio HiFi reads used to polish the genome and the final chromosome scale genome assembly showing full haploid collapsing of the chromosome-scale assembly. Colors in the key (top left) indicate the number of 27-mer copies in the genome assembly. **(e)** 3D-projection of stained DNA (magenta) in an *R. diutinus* nucleus from the transition zone exit region, a prometaphase I/metaphase I oocyte, and a prometaphase zygote. Pairs of homologous chromosomes (indicated with white lines) and individual chromosomes (indicated with white dashed lines) are arbitrarily numbered. Note that a "2n=10 chromosomes" karyotype was consistently observed at all these stages. n=number of transition zone nuclei/oocytes/embryo nuclei examined; N=number of embryos used (for embryo nuclei analysis only) and are indicated at the top of each image. Scale bar, 5 µm.

Supplementary Fig. 2

a

```
         1        10        20        30        40        50        60        70
CelSAS-4 M..ASDENIGADGEQKPSRPFLRKGQG.TARFRMPRNNKTSAGAPPTSELSSASSPSINVPRFSLSNALPNSARTVDSGI
RdiSAS-4 MDGEDNENVTPIKAEKPIQRFMRKGEGVTGKFK.....................GKINYPAIGLAKA..HSTPKQDDVV

          80        90       100       110       120       130       140       150
CelSAS-4 SNEDETRPPTTASLPMDQPSLSSSPENRLNPAPSVAEEHGHSGQHAEEEEDNDTDEVSAMPSFVPDEPSTLVNSDHELSD
RdiSAS-4 LDDYQTE.....SSNGEDVYIETLEAHAIRKQPLGSRSNGFGGTKYSDKGNNHGPS.SSESEIV..EPSAIA...HENTE

         160       170       180       190       200       210       220       230
CelSAS-4 DALKYKNAAAEFKAFERRMDSMRSASTITTS..LATPSSCAPSNSSEPPTRSTPIMNDLGVGPNNHNWPSSMQELSGISL
RdiSAS-4 D.........FEIAERMVQSARQPNFVVLEQPVVSPLGQRKSNLQEDGRR....FSRYNTMPRQIHTPSQMSS......

           240       250       260       270       280       290       300       310
CelSAS-4 ETPQARPLGSNRINQLVRSEAQTGISLLQHHERPTV.TAPLRRNDMMNSSRQNPQNGNVQDENRPEHVYDQPIHVPGSSL
RdiSAS-4 .......TGSNFTATIMQRN........NYFDRDSYSSSPSAIHGKINSLGSIPETETPRTKSSYRDRYDESQVNP....

             320       330       340       350       360       370       380
CelSAS-4 DRQKLEIEIRRHRNLNIQLRDTIAHLDYAEESVHTTKRQL....EEKISEVNNFKKELIEEFKKCKKGVEE...EFEKKF
RdiSAS-4 ..............LEDQLEHQILTIDYAFTQLKARMDMVFVVREKRLGEVEDDLKGQIMSFEQEKRIIHEKNTQYQKTI

         390       400       410       420       430       440       450       460
CelSAS-4 EKIKEDYDELYEKLKRDQRDLERDQKILKKGTGERNKEFTETIATLRDKLRASETKNAQYRQDIRVRD....EKLKKKDE
RdiSAS-4 QRLKEENEE..................LKKGNALAKQE....NETLKGKYLAASTKVSNIRTKLSTVEKEYKEEMVKKNE

                 470       480       490       500       510       520       530
CelSAS-4 EIEKLQKDGNRLKSTLQT...LEKRVKQLRTEKERDDKEKEM...FAKV.AMNRKTSNPVPPVLNQSVPISITSNGPSRH
RdiSAS-4 EIANLRKRLERKYSLNQTDPGYYNDAHQVGERRKRNSYPEEMNDGNDRIGTSSRRTSYGLNSSIKQSPTCCHHDNGHIEL

           540       550       560       570       580       590       600
CelSAS-4 PSSSSLTTFRKP..STSNRERGVSWADEPNEQSLEAVPQE....FLMMPVKEMPGKFGKCT.IYRDSLGETSKVTDTIAN
RdiSAS-4 SVPNKQVKWQNPVAETQAFETVVDSIMIDRDNSTGSVSHEEEINSHMKKIKFDINSDSDCVGVFSDKRSQTVYLSKN.CG

      610       620       630       640       650       660       670       680
CelSAS-4 GLLFEYSNGDLRWVNRQNAVNIYISAVDKTVRIDLPTYNISIIHTFQRQVEVLRPGNNITLISIKRREVRTDLIYQ.NGM
RdiSAS-4 CQVYKYSNGDRRWVHSDSVYELYYMALEGTNIVSIMDEWTILFQSGTGQLEISRSDGQ..KIVINRDGVRTDLRKSTDGT

         690       700       710       720       730       740       750       760
CelSAS-4 YKTEIF.NRDGRYVTKDFSNQEVSRKYNPGTHTYRDNQCRYVLVTDYNDFELVEPEFRLRWYQGDPTGLNNQYILKIIGR
RdiSAS-4 YIAEIYKGRKKVTVTSITLKTEIKDTKNP..VLWKDSRMEH...GENNIYRFETSSFKL...NRSPEGQ.....VKIILM

         770       780       790       800
CelSAS-4 PECSEKTLRLEVNLSTCEGTLETAEMIGDKRRKTTLFQWKK...............
RdiSAS-4 LVQTKDEISLLVEDVTC...VKHVTKKNDITIKETCLLWGKSAKAVVPIPVNTGNS
```

b

```
         1        10        20        30        40        50        60        70        80
CelTBG-1 MSGTGALMTVHVGQCGNQLAQAFWKSMVDEHGINERGQTTHEDDMNDKKDLLFYQADDDHYVPRAVLVDLEPRVINGMMQ
RdiTBG-1 M..PGEIINVQLGQCGNQIGMEFWRTLCAEHGIMPDGSFNHEETGTDIKDIFFYQADDRRFVPRAVLIDLEPRVIKTIM.

                  90       100       110       120       130       140       150       160
CelTBG-1 SPNFSNLFNTDNIFMSDHGGGAGNNWASGYCQGQEVQEKIMDIIIREAENTNNLDGILFTHSVSGGTGSGTGSLLLERLR
RdiTBG-1 SGDYKRFFNHENVFTSQNGGGAGNNWANGYLQGEEYIESVLDILDREAEFSENLDGFMLCHSIAGGTGSGMGSYVLEKIK

                 170       180       190       200       210       220       230       240
CelTBG-1 EAFPKKVIQTYSVFANSDTSTDVVVHPYNWVLSMQRLIENPDHVVVLDNAALHRLAAGKFKTDTPTFDHINSLVARIMST
RdiTBG-1 EKYPKKILKTYSVFPNQQETSDVVVQPYNSILSLERLIENPDAVTVLDNTQLDRLAVEKMHVDKPTFTQINSMVSRIMSA

                 250       260       270       280       290       300       310
CelTBG-1 STAPYRFNSAMCPSIRYL..DLAPFPPMHFIQSAISPVVDPNENFTRKTSVADVTRFLLKPTSMMVSTASRVRPNDCMLS
RdiTBG-1 ATSTMRFPTSTHTRLSSIISRLVPFTPMHFIQTGFTPLC..SDQFVVRTSVQDVMRRLVQPGSMMVNTRIDKKTDHCMLG

       320       330       340       350       360       370       380       390
CelTBG-1 AYMFLQGQIEAHTIMTAEQNVD..FAIRRPPFYMLKPLRMMHAPLSPYVRPQYKVSGLLLNNSTSVAPLFESLLSKYDKL
RdiTBG-1 GLVIFQGEADPKDVSDAFDRIQQKRSIKAPPYFPI.PLEVAIAGRSPYVTTTHKVSGLLMGNNTNICQLFEHTLQQFDKL

           400       410       420       430       440
CelTBG-1 RSKRAFIDKFEKI.DNFSLDM.MDDAMHIVQDLLDEYKAVVQKDYLTR............GL
RdiTBG-1 YKNNAFINNFTKISDDFDIKYHLENAREKVHNLCDMYRAATKADFMNMTCSGQDWSDTVDTV
```

Supplementary Figure 2: Identification of *R. diutinus* orthologs of SAS-4$^{\mathrm{CENPJ/CPAP}}$ and TBG-1$^{\gamma\text{-tubulin}}$. Amino acid sequence alignment between **(a)** *C. elegans* (*Cel*) and *R. diutinus* (*Rdi*) SAS-4 and **(b)** *Cel*TGB-1 and *Rdi*TBG-1. Identities are highlighted in orange, similarities are in orange font. The immunogenic peptides used for antibody production are squared in black.

Supplementary Fig. 3

a

```
1         10        20        30        40        50        60        70
CelZYG-1  MSGGKSGSRLSAYSHLKEIGKGGFGVVYAAQRENGEK.VAIKRIDK.KVPKNRVWTEIQTMKELKKSKYVVEFYEDFVED
RdiZYG-1  MT.ELVESR..GYKLIRSIGKGGFAECYAAVRNKDKKDVAIKIIKKEKQNKKLIDAELKAHKTFKHSCF.VEYYDDFGDK

80        90        100       110       120       130       140       150
CelZYG-1  GYTYIVMELCEGGSLQAYVREHGALDDATAVHVLRQLISAVSFMHRVNVIHRDLSAGNVFI.KDSKKKKMTVKLGDFGLA
RdiZYG-1  EVHCIVMELCSHGSLKEHIRTNGKLREDQAKSVLEKLIRAVSILNDNNVMHRDLSTGNILISRWMKGGDLVVKLADFGLV

160       170       180       190       200       210       220       230
CelZYG-1  TTLGRGETTCTIVGTPGFIAPQVYDQEYTQSADVYSLGAVLYTMLTARNPP.PKG.....LPPTCGMSPNAARLVEQMMD
RdiZYG-1  KFMDASKNCNTIAGTPHFIAREVMDGEYDKKADVFSLGCVLFAMLTAKPPPIDEGNDILRIVKRASLSTDATDLLKMLD

240       250       260       270       280       290       300
CelZYG-1  TDAKKRIPLTQIVLSEFMYENTNENAVIFSREHSRDGRRQRSREPVRSSRDDRSRDGRALIRSSSQPAHSGRAPLSN...
RdiZYG-1  .KKERRIELKHILKEPFICGCGRLSEVVSAR......RRSASRE.....EGNNGHKSVTSIRSVTRPPIEKNSDILNIIE

310       320       330       340       350       360       370       380
CelZYG-1  ..RPIHDRMPSTSSRGFDSERGR.....ERDRDSGRGTVPPSREDRNRSQLWPIRMDRLEGQRVCTAGGRYIVEL.DTRC
RdiZYG-1  NGTSVESMMSTRKPRSIRKSNGLSSGYVSNATDCTKVTI..ASKLRREKMTWPLPLFSLTNIVIAVGKQKFTVEMRDDDC

390       400       410       420       430       440       450
CelZYG-1  RFEVAAQGNFVKRILIVEVDEMVQTVYV.....HRIPDRTVRGRNGEEELITLTNNPFVY..TSYSQMPKEVQN.DYMRL
RdiZYG-1  LLQ.CEFGN........QNDELIGAMIVKQRIRHSKTTQTIEFYNLKKSNCDQSFNEFVHLEEKWLKDPTVFKNLDGLKC

460       470       480       490       500       510       520
CelZYG-1  Q.......KMV...AVTISGRVAKVTFRRPSQFPDAQAQLMENGDLRIKLP.RSVIVRKMDNGEIFNCIDGIATQKQAVS
RdiZYG-1  SGRPLTAYKIVHDFVFNMLARVEKARFNNTGGIRGAISRFMANGDIKVTMPDKRVGILKY..GE..TSFKSLENKRCPFN

530       540       550       560       570       580
CelZYG-1  GITLTKVNEVYKYLIRFEQCLNGMDRGMV......CFPI...VFSAGTNMVG..........SSPSSLMPS.GSSQTSRF
RdiZYG-1  ESELYKLRKISHQFEAWEEA..GKDHEEVNMEESSCHILDDDSYSVKRNTSGKIVQIIVQKDKVKKSLRESTGDSNTYVY

590       600       610       620       630       640       650
CelZYG-1  PFSNLSNNQPSLVPHSAPFLTKPTSSQRASSANVQRRVSTDENSSPSVAPSKYKIKIDPT..TQKVRSIQATDGRV....
RdiZYG-1  TKSGSGEHRFVFKGGSMNAVRRDVRLLLSTLLHCQKNDSTKEND....ALAKENVRFGSTSKTGPINSIKLVGNKFKCAT

660       670       680       690       700
CelZYG-1  .LRCSTSKADQFIFTDPAIRPDDQRFMRTDRVPDRAS...EMLHTLCERMRKLHQ.
RdiZYG-1  NLRISGSNMNQFVHKDRHGEYKRVNFGENDRLSEVDSFYRPVLKLLEEMGRSYK
```

b

```
1         10        20        30        40        50        60        70
CelAIR-1  MS...GKEN.TAPVIDDQKAEVISLTEDSRPQRVDQAREESCWSLDDFDVGRPLGKGKFGNVFISREKKTKRIIALKVLF
RdiAIR-1  MSEAVGKENHLSPV.....SEELEILEKDGTKR.......KMWVLDDFEIGRCLGKGKFGNVYLAREKHSRFVVALKVLF

80        90        100       110       120       130       140       150
CelAIR-1  KTQLLQLGVSHQLKREIEIQYHLRHPNILTLYGYFHDDKRVFVILDYASRGELFNVLQSQPGHKVNEVIAGRFVRQLANA
RdiAIR-1  KEQIEKFQVRVQIKREIEIQFHLRHQNILRLYGYFYDNLRIYLILEYASRGTLFSYLQSK..KTLKNSVAAKYLFQLTDA

160       170       180       190       200       210       220       230
CelAIR-1  LHYCHSKGVIHRDIKPENLLLDSKLNLKLADFGWSVVADHSKRHTLCGTMDYLAPEMVSNQ...PHDFNVDIWAIGILLF
RdiAIR-1  LDYCHNLNVIHRDIKPENLIISAKDDLKIADFGWSVYTPDSRRQTVCGTLDYLPPEMIGGKDKGTYDYTVDNWAVGVLLY

240       250       260       270       280       290       300       310
CelAIR-1  EMLVGYAPFANQTGDKLIARIKECKIYIPSVVTDGAASLINAIIKKEPQERLPLVDIMAHPWIKEMKQREDIEVPLFIST
RdiAIR-1  EMLVGKPPFEQEDQTDTLKSIAKASYTIPEFVPQDASWIINSLLKAIPSERVSLDEVKKSAYI..................

320
CelAIR-1  LTKSSRNNSTANQ
RdiAIR-1  .TK....NYVNS
```

Supplementary Figure 3: Identification of *R. diutinus* orthologs of ZYG-1^{Plk4} and AIR-1^{AuroraA}. Protein alignment between **(a)** *Cel*ZYG-1 and *Rdi*ZYG-1 and **(b)** *Cel*AIR-1 and *Rdi*AIR-1. Identities are highlighted in orange, similarities are in orange font. Kinase domains of *C. elegans* AIR-1 and ZYG-1 are underlined in light blue. The immunogenic peptides used for antibody production are squared in black.

Supplementary Fig. 4

a

	Peptide 1	Peptide 2
***Rdi* SAS-4**	CQRNNYFHRDSYSSSP	CKRNSYPEEMNDGNDR
***Rdi* TBG-1**	CSGQDWSDTVDTV	
***Rdi* ZYG-1**	CQKNDSTKENDALAK	
***Rdi* AIR-1**	MSEAVGKENHLSPC	CKSAYITKNYVNS

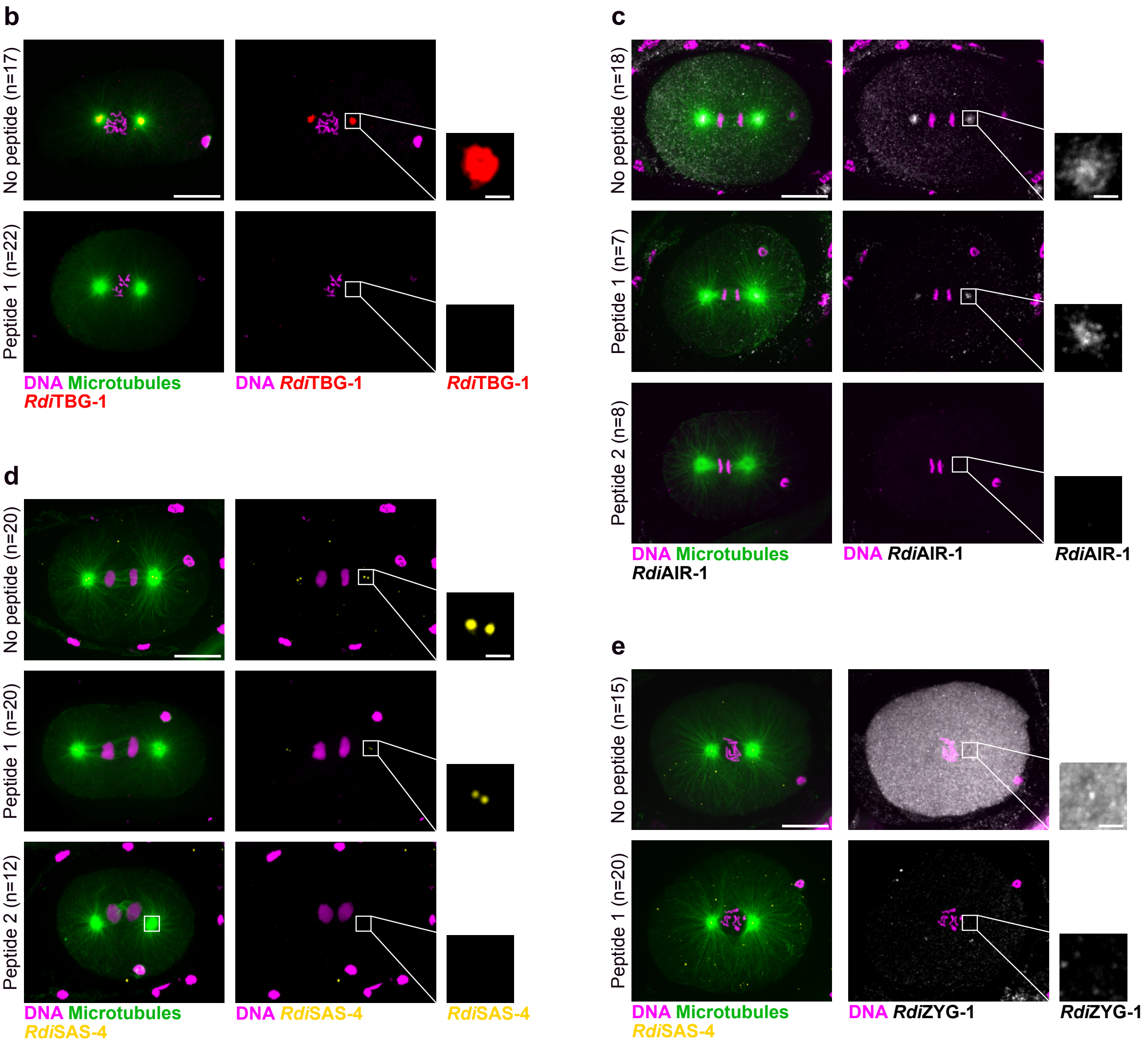

Supplementary Figure 4: Validation of *R. diutinus* antibody specificity. (a) List of antigenic peptides used for the production of antibodies to *R. diutinus* proteins. **(b-e)** Immunofluorescence images of *R. diutinus* embryos after antibody incubation with or without the corresponding immunogenic peptide. Number of spindles examined is indicated on the left for each condition. Magenta, DNA; green microtubules (**(b-e)**, left panels); red, *Rdi*TBG-1 **(b)**; grayscale, *Rdi*AIR-1 (**(c)**, right panels) or *Rdi*ZYG-1 (**(e)**, right panels); yellow, *Rdi*SAS-4 (**(d)**, all panels; **(e)**, left panels). Scale bar, 10 μm. Insets show higher magnification view of *Rdi*TBG-1, *Rdi*AIR-1, *Rdi*ZYG-1, and *Rdi*SAS-4 at the indicated mitotic spindle pole. Scale bar, 1 μm.

Supplementary Fig. 5

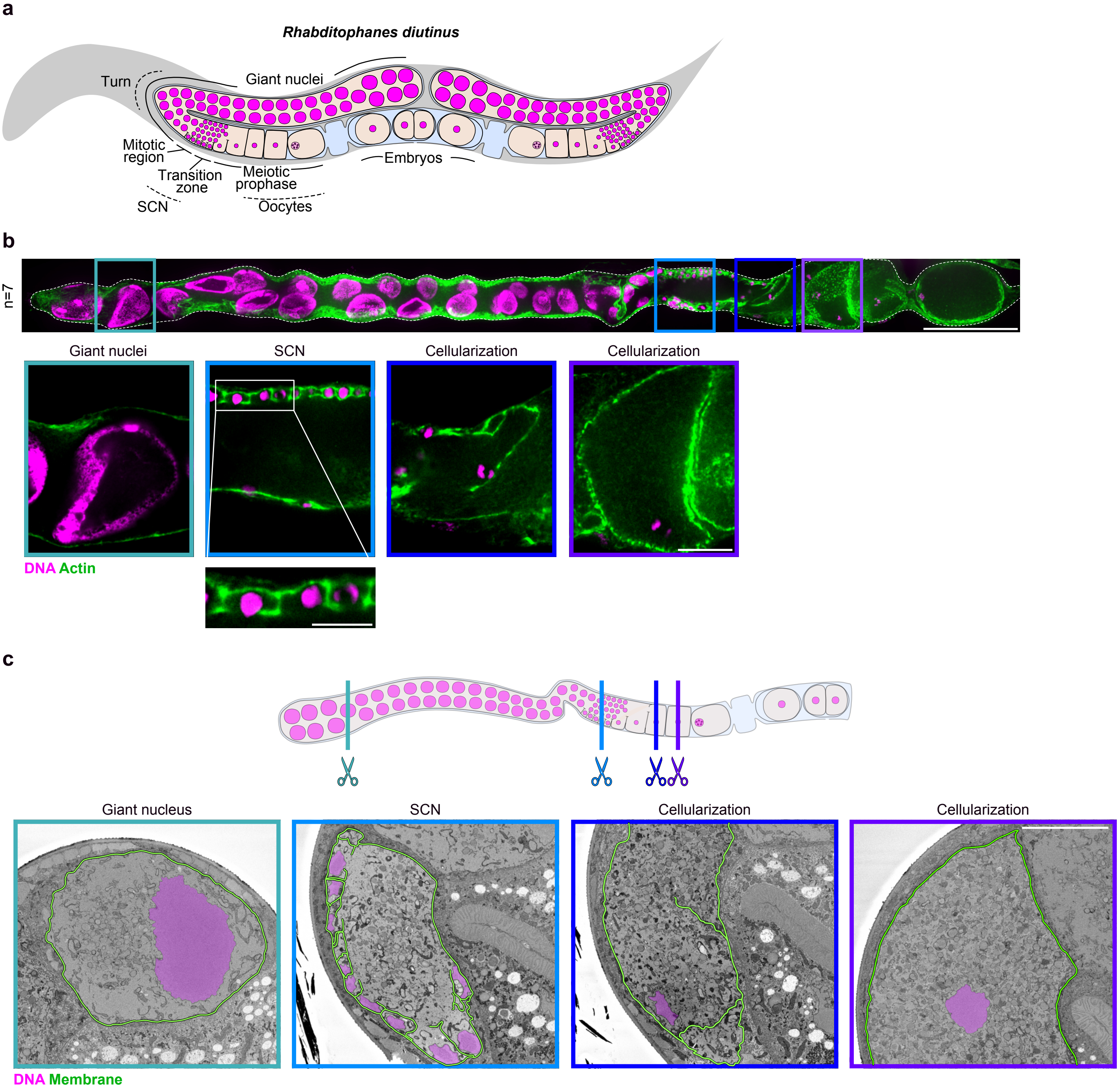

Supplementary Figure 5: *R. diutinus* gonad organization differs from that of *C. elegans*. (a) Schematic of an adult *R. diutinus* highlighting the reproductive system. Magenta, DNA. **(b)** Phalloidin staining of a straightened *R. diutinus* gonad. Magenta, DNA; green, F-actin. Number of gonads examined is indicated on the left. Scale bar, 50 µm. Bottom insets show higher magnification views of regions of interest. Scale bar, 10 µm. Scale bar of the SCN region bottom inset, 5 µm. **(c)** Serial Block-Face Scanning Electron Microscopy (SBF-SEM) of an *R. diutinus* gonad at four slices of interest. (Top) Schematic of a straightened *R. diutinus* gonad showing the position of the slices of interest along the distal-proximal axis. (Bottom) SBF-SEM images of the indicated slices of interest. Magenta, DNA; green, plasma membrane. Scale bar, 10 µm.

Supplementary Fig. 6

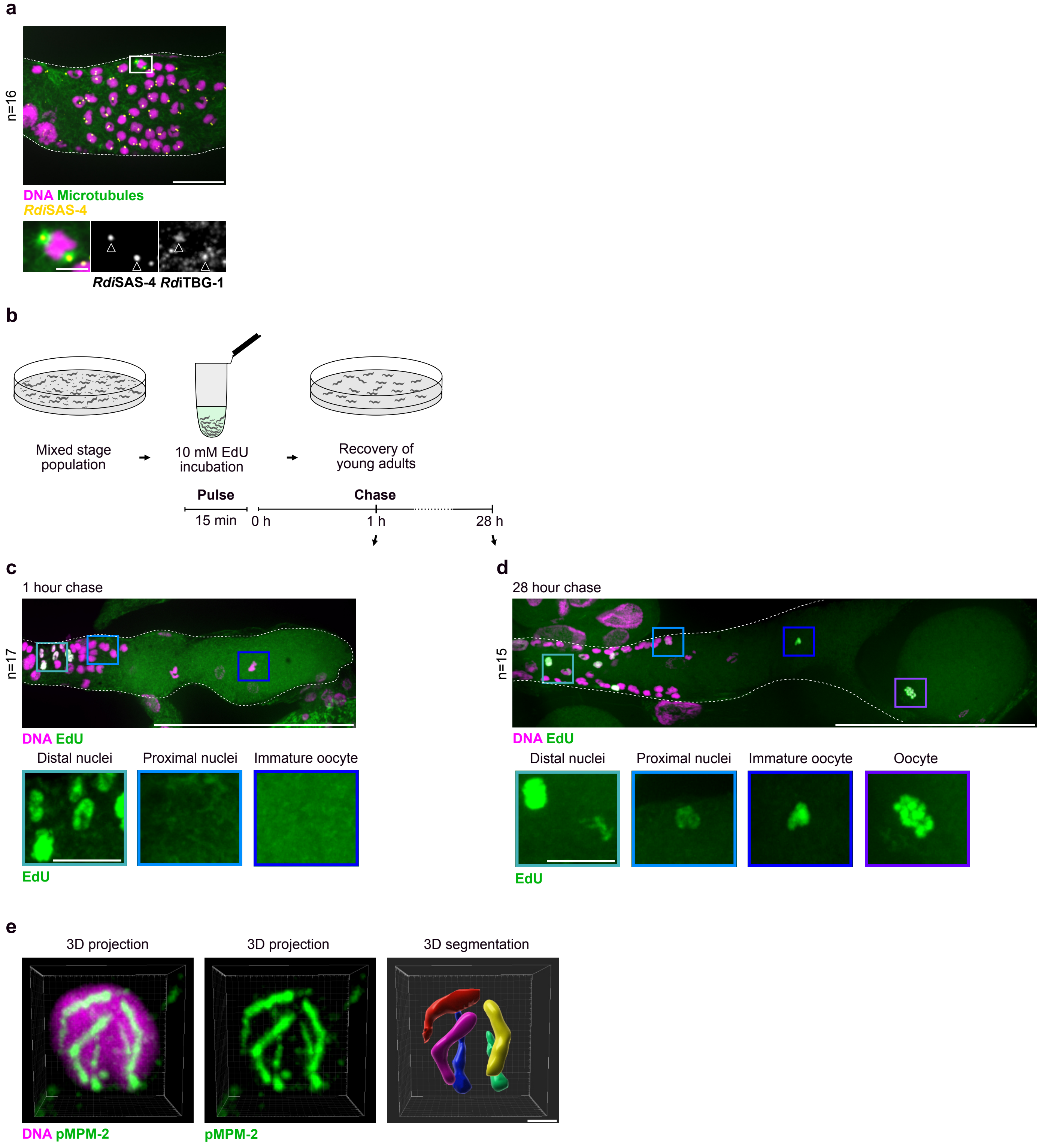

Supplementary Figure 6: Identification of germline stem nuclei in *R. diutinus*. (a) Immunofluorescence image of a fixed *R. diutinus* gonad centered on the spermatheca-proximal region, which contains the SCN and stained for microtubules (green), DNA (magenta), and *Rdi*SAS-4 (yellow). Number of gonads examined is indicated to the left of each image. Scale bar, 10 µm. Bottom inset show higher magnification view of single spindle with indicated staining. Black arrowheads indicate *Rdi*SAS-4 and TBG-1 signals. Scale bar, 2 µm. **(b)** Schematic of experimental strategy for EdU incorporation pulse-chase experiment. **(c-d)** Representative images of *R. diutinus* gonads pulsed with EdU, after a 1-hour **(c)** or 28-hour **(d)** chase. Magenta, DNA; green, EdU signal. Scale bar, 50 µm. Bottom insets show higher magnifications of regions of interest. Scale bar, 5 µm. **(e)** 3D-reconstructions of an early meiotic *R. diutinus* nucleus stained with an anti-pMPM-2 antibody. Scale bar, 1 µm.

Supplementary Fig. 7

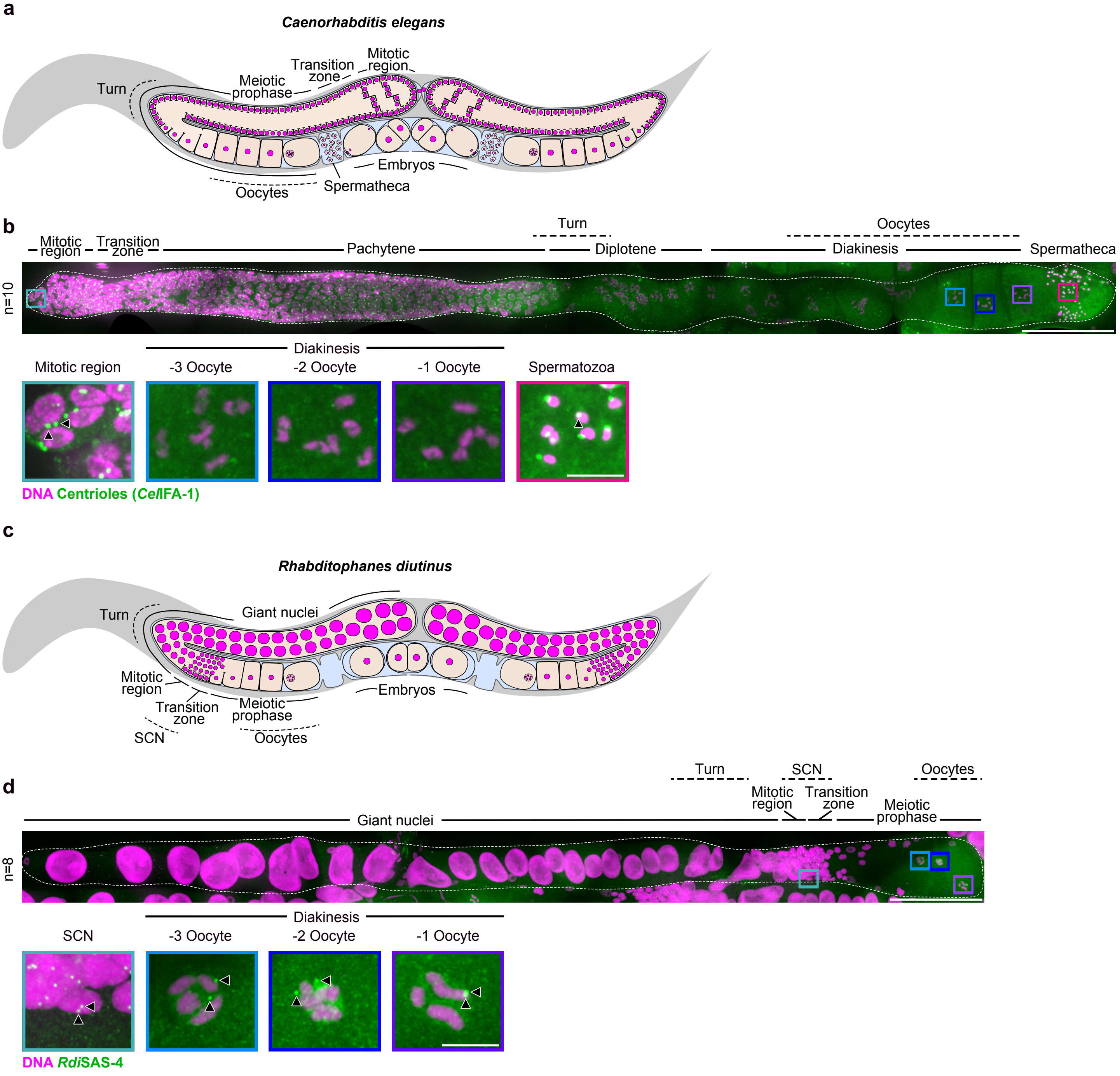

Supplementary Figure 7: Centrioles are eliminated by diplotene in *C. elegans* but maintained in the germline of *R. diutinus*. (a,c) Schematic of the adult hermaphrodite *C. elegans* **(a)** or female *R. diutinus* **(c)** reproductive systems. Magenta, DNA. **(b, d)** Immunofluorescence images of a straightened *C. elegans* **(b)** and *R. diutinus* **(d)** gonad stained for the centriolar markers **(b)** *Cel*IFA-1 (green) or **(d)** *Rdi*SAS-4 (green), respectively. Number of gonads examined is indicated to the left. Magenta, DNA. Scale bars, 50 µm. Bottom insets show higher magnification views of regions of interest. Black arrowheads indicate centriolar foci. Scale bars, 5 µm.

Supplementary Fig. 8

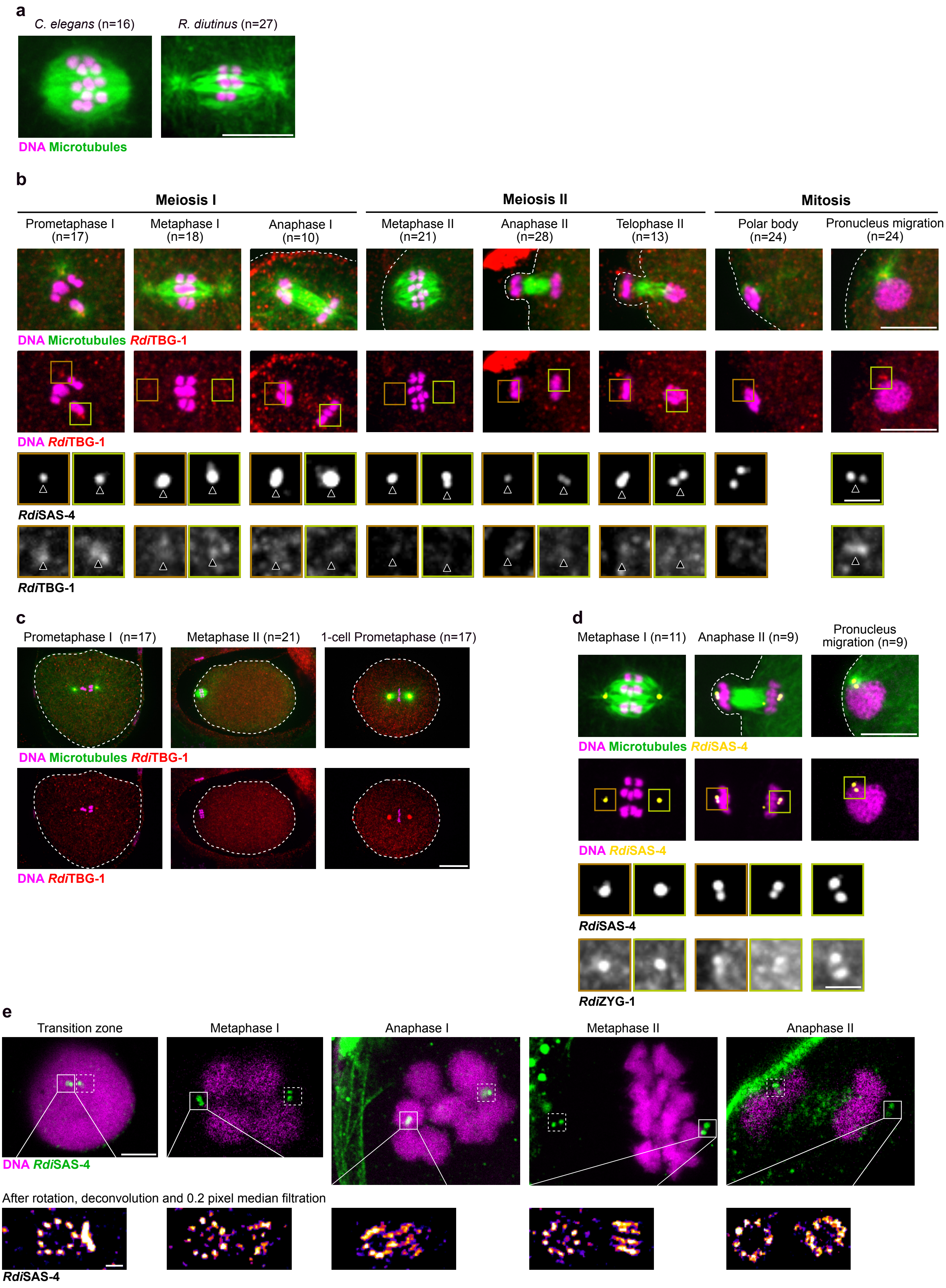

Supplementary Figure 8: Centriole maintenance throughout meiosis in *R. diutinus*. (a) Immunofluorescence images comparing *C. elegans* (left) and *R. diutinus* (right) oocyte meiotic spindles in meiosis I. Number of oocytes examined is indicated at the top of each image. Magenta, DNA; green, microtubules. Scale bar, 5 µm. **(b)** Immunofluorescence images of the spindle at indicated meiotic division stages, and of the polar body and zygotic maternal pronucleus in *R. diutinus*. Magenta, DNA; green, microtubules; red, *Rdi*TBG-1. Scale bars, 5 µm. Oocyte and embryo contours are highlighted with a dashed line. Number of oocytes or embryos examined is indicated at the top for each stage. Bottom insets show higher magnification views of *Rdi*SAS-4 and *Rdi*TBG-1 at the left (brown squares) and right (green squares) meiotic spindle poles, in the polar body (brown squares), and during pronuclear migration (green squares). Scale bar, 1 µm. **(c)** Immunofluorescence images of *R. diutinus* prometaphase I and metaphase II oocytes and one-cell embryos. Magenta, DNA; green, microtubules; red, *Rdi*TBG-1. Oocyte and embryo contours highlighted with a dashed line. Number of oocytes and embryos examined is indicated at the top for each stage. Scale bar, 10 µm. **(d)** Immunofluorescence images of the spindle at the indicated oocyte meiotic division stages, and of the polar body and maternal pronucleus in *R. diutinus*. Magenta, DNA; green, microtubules; yellow, *Rdi*SAS-4. Scale bar, 5 µm. Oocyte and embryo contours are highlighted with a dashed line. Number of oocytes and embryos examined is indicated at the top for each stage. Bottom insets show higher magnification views of *Rdi*SAS-4 and *Rdi*ZYG-1 at the left (brown squares) and right (green squares) meiotic spindle poles and during pronuclear migration (green squares). Scale bar, 1 µm. **(e)** U-Ex-STED images of centrioles observed at *R. diutinus* oocyte spindle poles at different meiotic stages. Magenta, DNA; green, *Rdi*SAS-4. Scale bar, 5 µm. Bottom insets show corresponding STED centriole images at one meiotic spindle pole, after rotation, deconvolution and a 0.2-pixel median filter. Centrioles located at the other pole are highlighted with a dashed white square. *Rdi*SAS-4, fire lookup table. Scale bar 0.2 µm.

Supplementary Fig. 9

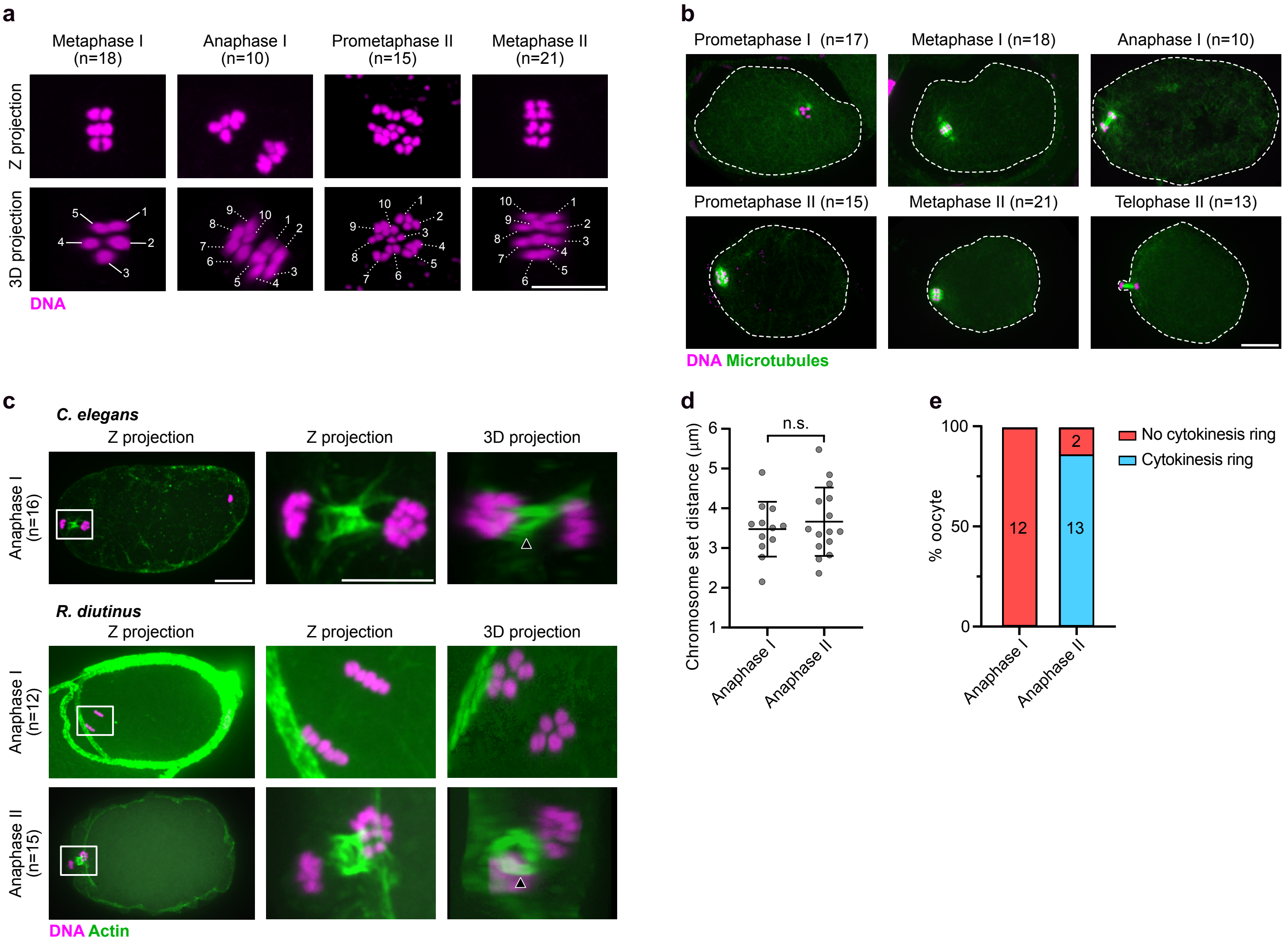

Supplementary Figure 9: Abortive cytokinesis at the end of meiosis I allows ploidy to be maintained in *R. diutinus*. (a) Staining of DNA (magenta) in *R. diutinus* oocytes at indicated meiotic stages. Z- (top) and 3D- (bottom) projections marking the five pairs of homologous chromosomes in metaphase I (chromosomes are arbitrarily numbered and indicated with white lines), and the ten pairs of sister chromatids during and after anaphase I and during meiosis II (pairs of sister chromatids are arbitrarily numbered and indicated with white dashed line). Scale bar, 5 µm. Number of oocytes examined is indicated at the top for each stage. **(b)** Full views of the *R. diutinus* oocytes displayed in Fig. 3b. Magenta, DNA; green, microtubules. Oocyte contours are highlighted with a dashed line. Scale bar, 10 µm. Number of oocytes examined is indicated at the top for each stage. **(c)** F-actin (fluorescent phalloidin) staining of a *C. elegans* anaphase I oocyte (top) and *R. diutinus* anaphase I (middle) and anaphase II (bottom) oocytes. Magenta, DNA; green, F-actin. Scale bar, 10 µm. Insets on the right show higher magnification views of regions of interest. Displayed are Z- (middle) and 3D- (right) projections. Black arrowheads indicate F-actin cytokinetic rings. Scale bar, 5 µm. Number of oocytes examined is indicated on the left. **(d)** Quantification of the distance between the segregating chromosomes in *R. diutinus* oocytes at the indicated meiotic stage. The mean and standard deviation are indicated. Two-tailed unpaired t-test, alpha=0.05, p-value=0.5456 (n.s.). **(e)** Percentage of *R. diutinus* oocytes with (blue) or without (red) F-actin cytokinetic ring at the indicated meiotic stages. The corresponding number of oocytes is indicated in each bar section. Source data are provided as a Source Data file.

Supplementary Fig. 10

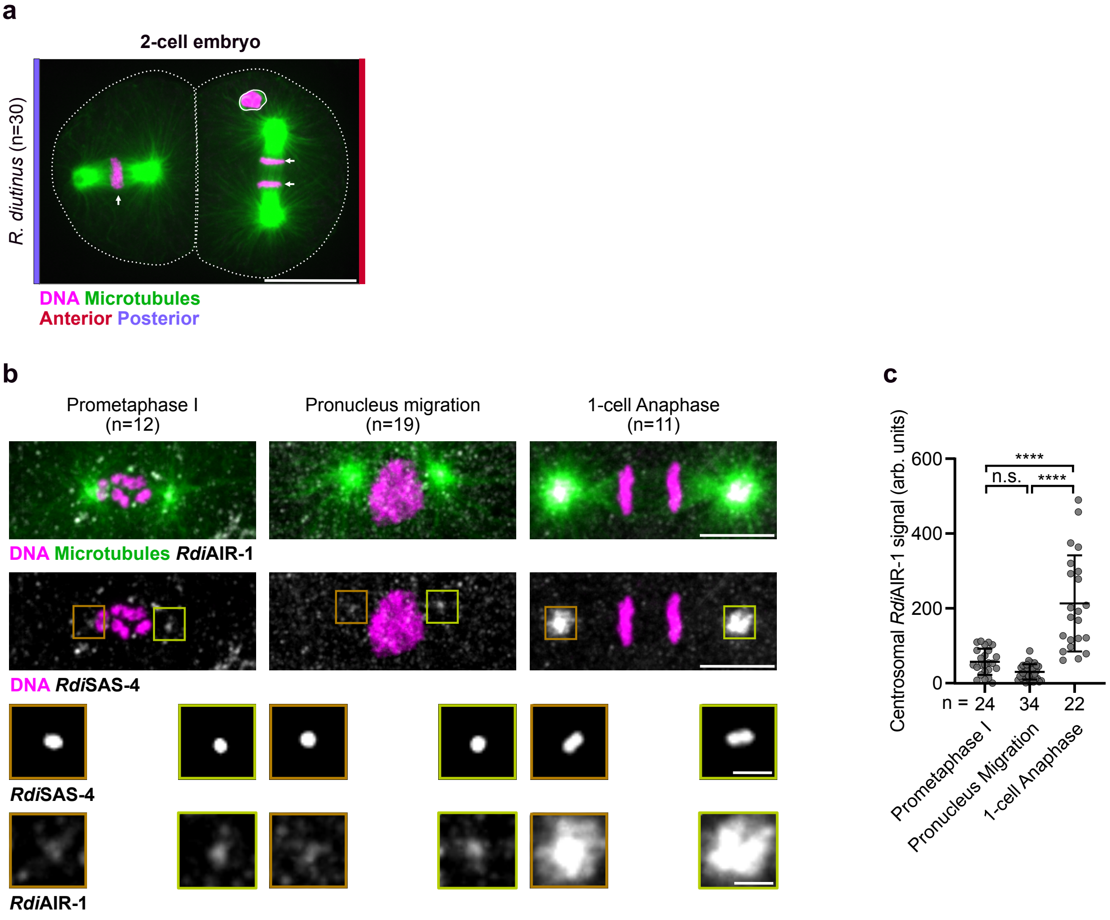

Supplementary Figure 10: Anterior-posterior polarity in the *R. diutinus* embryo. (a) Immunofluorescence image of a two-cell stage *R. diutinus* embryo highlighting the offset timing of division between the anterior AB and the posterior P1 blastomere. The blastomere and polar body contours are highlighted with a dashed and a straight line, respectively. The white arrows indicate the aligned metaphase chromosomes in the P blastomere and the segregating anaphase chromosomes in AB. Magenta, DNA; green, microtubules. Scale bar, 10 µm. Number of embryos examined is indicated on the left. **(b)** Top, immunofluorescence images of *R. diutinus* embryos centered on the spindle region stained for *Rdi*AIR-1 at the indicated stages**.** Magenta, DNA; green, microtubule; greyscale, *Rdi*AIR-1. Scale bar, 5 µm. Number of oocytes or embryos examined is indicated on the top for each stage. Bottom insets show higher magnifications of *Rdi*SAS-4 or *Rdi*AIR-1 at the left (brown square) and right (green square) spindle poles. Scale bar, 1 µm. **(c)** Quantification of *Rdi*AIR-1 centrosomal signal at the indicated stages. One-way unpaired ANOVA test, alpha=0.05, ****=p≤0.0001. Mean and standard deviation are displayed. Number of oocytes or embryos examined is indicated below the plot for each stage. Source data are provided as a Source Data file.
